# Oxeiptosis: a novel pathway of melanocytes death in response to oxidative stress in vitiligo

**DOI:** 10.1038/s41420-022-00863-3

**Published:** 2022-02-17

**Authors:** Pan Kang, Jianru Chen, Weigang Zhang, Ningning Guo, Xiuli Yi, Tingting Cui, Jiaxi Chen, Yuqi Yang, Yinghan Wang, Pengran Du, Zhubiao Ye, Baizhang Li, Chunying Li, Shuli Li

**Affiliations:** Department of Dermatology, Xijing Hospital, Fourth Military Medical University, No. 127 of West Changle Road, Xi’an, Shaanxi 710032 China

**Keywords:** Chronic inflammation, Cell death

## Abstract

Vitiligo is a cutaneous depigmenting autoimmune disease caused by the extensive destruction of epidermal melanocytes. Convincing data has defined a critical role for oxidative stress in the pathogenesis of vitiligo. Oxeiptosis is a caspase-independent cell death modality that was reportedly triggered by oxidative stress and operative in pathogen clearance. However, whether oxeiptosis exists in oxidative stress-induced melanocytes demise in vitiligo remains undetermined. In the present study, we initially found that other cell death modalities might exist in addition to the well-recognized apoptosis and necroptosis in H_2_O_2_-treated melanocytes. Furthermore, AIFM1 was found to be dephosphorylated at Ser116 in oxidative stress-induced melanocytes death, which was specific to oxeiptosis. Moreover, KEAP1 and PGAM5, upstream of the AIFM1 in oxeiptosis, were found to operate in melanocytic death. Subsequently, the KEAP1-PGAM5-AIFM1 signaling pathway was proved to be involved in oxidative stress-triggered melanocytes demise through the depletion of KEAP1 and PGAM5. Altogether, our study indicated that oxeiptosis might occur in melanocytes death under oxidative stress and contribute to the pathogenesis of vitiligo.

## Introduction

Vitiligo is an autoimmune skin disease characterized by chronic depigmentation and milk-white lesions, which result from the destruction of epidermal melanocytes [1]. Oxidative stress is one of the most fundamental factors triggering melanocytes destruction in vitiligo pathogenesis [2]. Previous studies have elucidated various pathways of oxidative stress-induced melanocytes death, like aberrant mitochondrial function, impairment of the antioxidant defense system, and release of cytokines associated with immune reaction activation [3–7], but the modality of melanocytes death was confined to apoptosis and necroptosis. Whether melanocytes undergo other death modalities remains to be further defined.

Pathological reactive oxygen species (ROS) accumulation causes irreversible conformation changes of macromolecules like protein, lipid, and DNA in cells with consequent structural and functional anomalies in organelles like mitochondria and endoplasmic reticulum, and finally the loss of cellular integrity [8–11]. But little was known about the oxidative stress-specific signaling pathways that lead to cell death until oxeiptosis was introduced. Oxeiptosis is recently described as a caspase-independent, apoptosis-like, ROS-induced cell death modality [12]. Oxeiptosis is found in mouse airway cells exposed to ozone and HeLa cells infected by influenza A virus, both of which instigate exogenous oxidative stress [13]. Oxeiptosis is mediated by Kelch-like ECH-associated protein 1 (KEAP1)-Phosphoglycerate mutase family member 5 (PGAM5)-Apoptosis-inducing factor mitochondrion-associated 1 (AIFM1) pathway [14].

KEAP1 is an important sensor for oxidative stress, it bears 27 cysteine residues in its C-terminus which can be modified in a ROS-dependent manner, conferring KEAP1 to sense and quantify the intracellular ROS levels and to give specific responses [15]. KEAP1 mediates the ubiquitination of nuclear factor, E2-related factor 2 (Nrf2), and repression of Nrf2-dependent gene expression under basal unstressed conditions [16]. The moderate concentration of intracellular ROS might modify the KEAP1 cysteine residues and induce decreased ubiquitination of Nrf2, thus the steady-state levels of Nrf2 are increased in the cell. Nrf2 then translocates into the nucleus and mediates the expression of anti-oxidative genes to protect the cell from oxidative stress. However, in the presence of high ROS concentrations, KEAP1 might be functionally switched from cytoprotective to death induction by disassociating with PGAM5 [12]. PGAM5 participates in the regulation of mitochondrial dynamics and programmed cell death like apoptosis and necroptosis through protein-protein interaction [17]. In the oxeiptosis, subsequent to the disassociation from the ternary complex, PGAM5 is internalized into the mitochondrial lumen to dephosphorylate its substrate AIFM1 at a highly conserved serine residue at position 116 [13]. AIFM is implicated in several death modalities like apoptosis and parthanatos through shuttling to the nucleus and promoting DNA degradation to induce chromatin condensation [18]. But in oxeiptosis, however, AIFM1 is retained in the mitochondria with downstream signaling remaining elusive [14]. Whether oxeiptosis is conducive to oxidative stress-associated melanocytes demise in vitiligo merits more detailed analysis.

In the present study, we found that other oxidative stress-associated melanocytes death modalities still existed after the suppression of previously reported apoptosis and necroptosis by their specific inhibitors. Sequentially, in vitro assays indicated that H_2_O_2_ induced dephosphorylation of AIFM1 at Ser116, which was a molecular event specific to oxeiptosis, in melanocytes pretreated with Z-VAD-FMK (Z-VAD) and Necrostatin-1 (Nec-1). Furthermore, KEAP1 and PGAM5, which were in oxeiptosis signaling pathway, were detected to be upregulated in H_2_O_2_ treated melanocytes and vitiligo lesional skin biopsy. Meanwhile, the genetic intervention of KEAP1 and PGAM5 suggested their promotion of melanocytes oxeiptosis under oxidative stress. Taken together, our study elucidated that oxidative stress could induce melanocytes oxeiptosis in vitiligo.

## Results

### Caspase-independent death modalities exist in melanocytes under oxidative stress apart from apoptosis and necroptosis

**Fig. 1:**
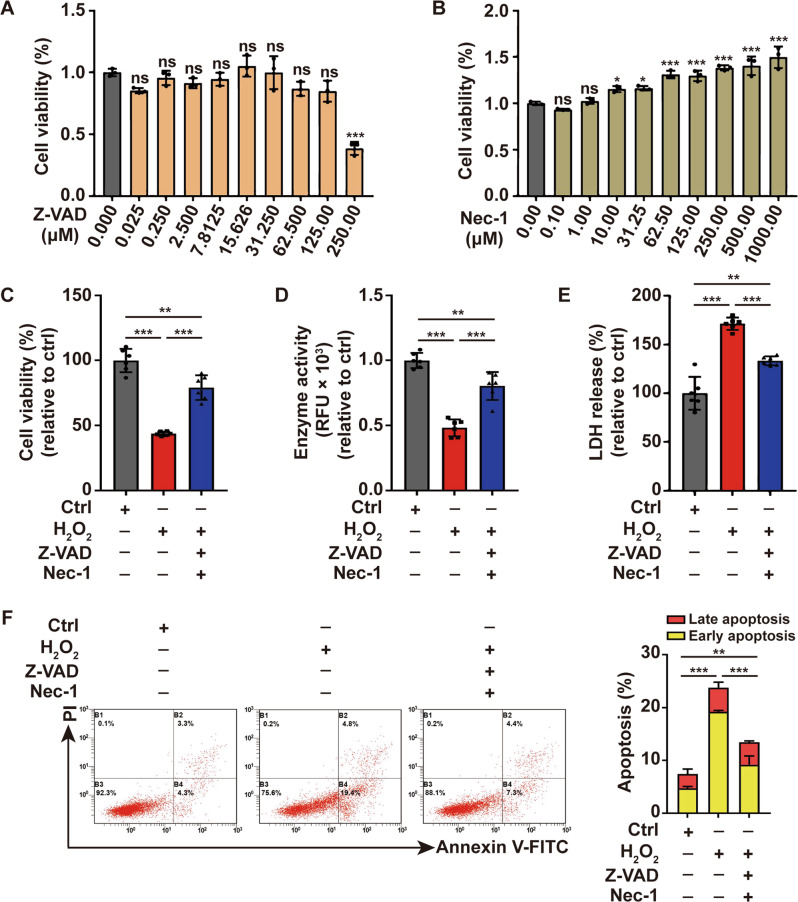
Oxidative stress induces caspase- and necroptosis-independent cell death in melanocytes. **A** The safety concentration of Z-VAD that does not affect cell viability in PIG1 cells was determined by CCK-8 assay. **B** The safety concentration of Nec-1 that does not affect cell viability in PIG1 cells was determined by CCK-8 assay. The cell viability of PIG1 cells treated by H_2_O_2_ or pretreated with Z-VAD and Nec-1 prior to H_2_O_2_ disposition is determined by CCK8 assay (**C**) and Resazurin-conversion assay (**D**). **E** The activity of released LDH in PIG1 cells treated by H_2_O_2_ or pretreated with Z-VAD and Nec-1 prior to H_2_O_2_ disposition was determined by LDH to release assay. **F** Annexin V-FITC/PI apoptosis assay of PIG1 cells pretreated with Z-VAD and Nec-1, followed by treatment with H_2_O_2_. ^*^P < 0.05, ^**^P < 0.01, ^***^P < 0.001, ns: not significant.

Previous studies have shown that oxidative stress could induce melanocytes apoptosis and necroptosis [3, 19, 20]. In order to investigate the involvement of other pathways in oxidative stress-induced melanocytes death, we detected survival of PIG1 in response to oxidative stress with pretreatment of caspase inhibitor Z-VAD and the necroptosis inhibitor Nec-1. First, we assayed which concentrations of Z-VAD and Nec-1 did not affect cell viability in PIGI cells by CCK8 assay. Nec-1 had no effect on cell viability at all concentrations tested, whereas Z-VAD reduced cell viability at concentrations exceeding 250 μM (Fig. 1A, B). According to previously published concentrations and assay times from the literature and our own viability data presented here, pretreating PIG1 cells with 20 μM Z-VAD and 40 μM Nec-1 for 1 h was selected in the following assay [21, 22]. Oxidative stress was induced by the treatment with 500 mM hydrogen peroxide (H_2_O_2_) as previously described [3]. The effectiveness of Z-VAD and Nec-1 was confirmed by decreasing the expression of cleaved-caspase3 and P-MLKL induced by H_2_O_2_ (Supplementary Fig. 1).

H_2_O_2_ treatment led to the decrease of cell survival as indicated by the lower cell viability and enzyme activity compared with control, which was detected by CCK8 assay and Resazurin-conversion assay (Fig. 1C, D). In addition, LDH release assay and apoptosis assay showed that LDH release and cell apoptosis increased in the H_2_O_2_-treated group (Fig. 1E, F), further confirming oxidative stress-induced melanocytes death. Importantly, pretreatment of PIG1 cells with Z-VAD and Nec-1 was not able to fully restore cell survival in response to the treatment of H_2_O_2_ (Fig. 1C–F). Collectively, these data suggested that apart from apoptosis and necroptosis, oxidative stress-induced melanocytes death could be mediated by other pathways.

### Oxidative stress induces dephosphorylation of AIFM1 at Ser116 in melanocytes

**Fig. 2:**
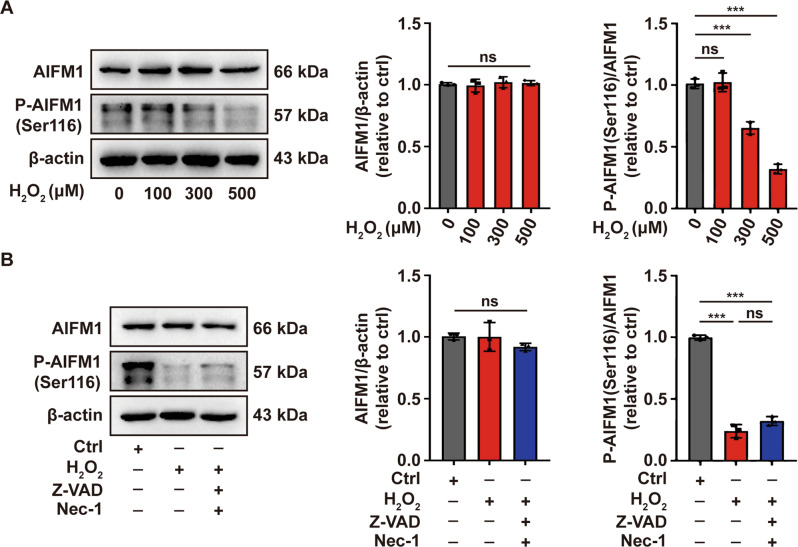
Oxidative stress induces dephosphorylation of AIFM1 at Ser116 in melanocytes. Western-blot assay of AIFM1 phosphorylated at Ser116 in PIG1 cells treated by the indicated concentration of H_2_O_2_ (**A**) or pretreated with Z-VAD and Nec-1, followed by treatment with H_2_O_2_ (**B**). ^***^P < 0.001, ns: not significant.

Oxeiptosis is a recently proposed caspase-independent cell death modality triggered by oxidative stress, which prompted us to investigate whether it occurs in melanocytes under oxidative stress. Dephosphorylation of AIFM1 at Ser116 serves as a marker and a fundamental molecular in oxeiptosis signaling pathway. Therefore, we initially detected the phosphorylation of AIFM1 at Ser116 in PIG1 cells treated with H_2_O_2_ by Western-blot assay. The results showed that AIFM1 was significantly dephosphorylated at Ser116 in PIG1 cells treated with 500 μM H_2_O_2_ (Fig. 2A). The combination of apoptosis- and necroptosis-inhibitors did not restore the dephosphorylation of AIFM Ser116 induced by H_2_O_2_ (Fig. 2B). These data collectively indicated that oxeiptosis might occur in melanocytes under oxidative stress.

### KEAP1 and PGAM5 participates in oxidative stress-induced melanocytes death

**Fig. 3:**
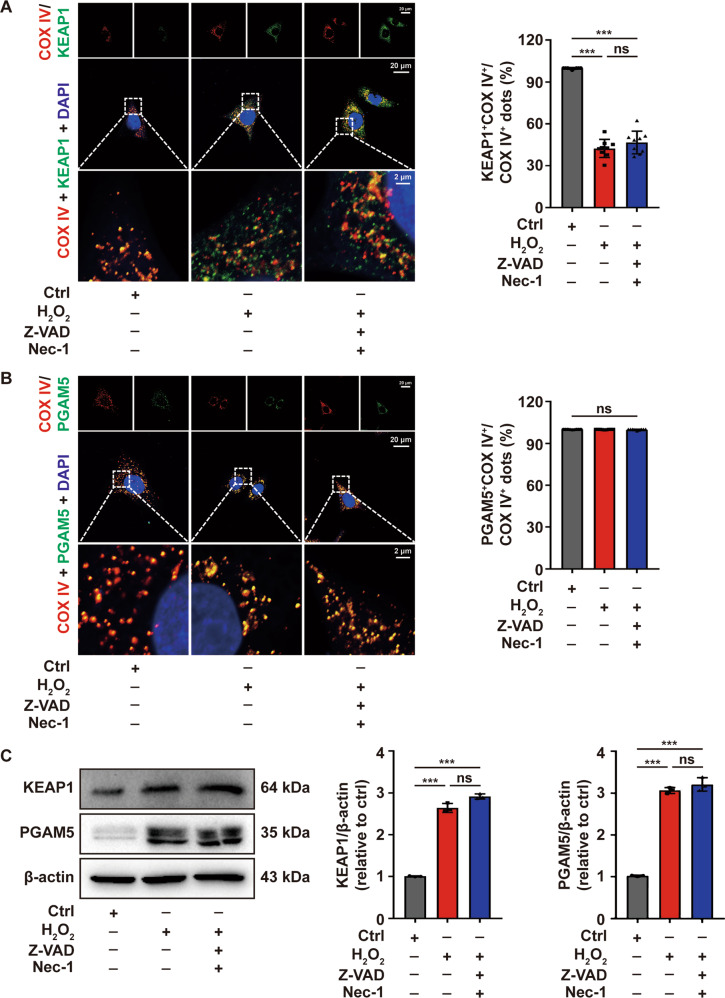
The expression of KEAP1 and PGAM5 were upregulated in melanocytes under oxidative stress. The location of KEAP1 (**A**) and PGAM5 (**B**) in PIG1 cells stimulated by H_2_O_2_ or pretreated with Z-VAD and Nec-1 prior to H_2_O_2_ disposition, detected by immunofluorescence assay. **C** The expression of KEAP1 and PGAM5 in PIG1 cells treated with H_2_O_2_ or pretreated with Z-VAD and Nec-1 prior to H_2_O_2_ disposition, detected by Western-blot assay. ^***^P < 0.001, ns: not significant.

As reported in the previous study, the oxeiptosis signaling pathway encompasses KEAP1-PGAM5-AIFM1 and the dissociation of PGAM5 from KEAP1 is a prerequisite for dephosphorylation of AIFM1. As indicated by immunofluorescence assay, KEAP1 and PGAM5 colocalized with mitochondrial marker COX IV in the control, while KEAP1 lost its colocalization with COX IV in the presence of H_2_O_2_, and pretreatment of PIG1 cells with Z-VAD and Nec-1 did not restore the dissociation of KEAP1 and COX IV induced by H_2_O_2_ (Fig. 3A, B), which further supported the putative oxeiptosis in melanocytes under oxidative stress.

**Fig. 4:**
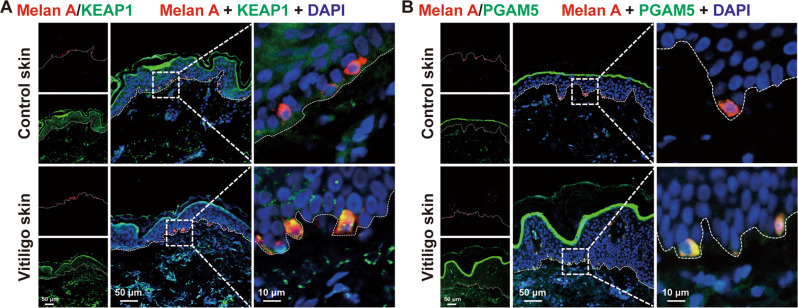
The expression of KEAP1 and PGAM5 were upregulated in melanocytes of vitiligo patients. The expression of KEAP1 (**A**) and PGAM5 (**B**) in vitiligo lesions and healthy control epidermis by immunofluorescence assay.

**Fig. 5:**
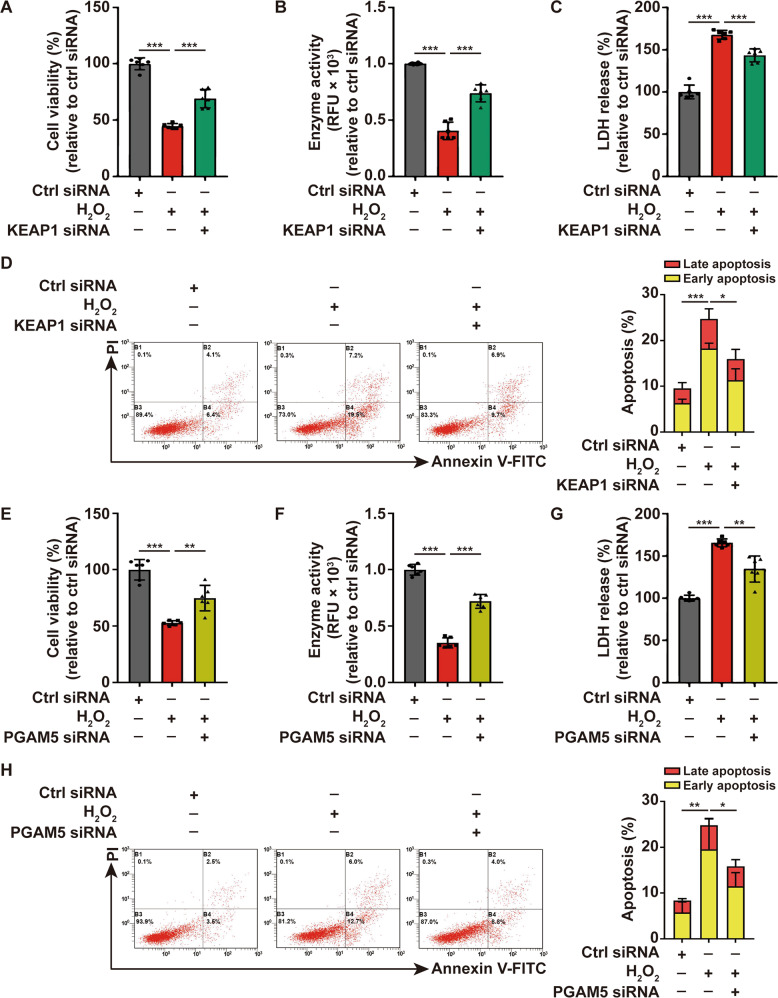
KEAP1 and PGAM5 participate in the cell death of melanocytes under oxidative stress. The cell viability of PIG1 cells treated by H_2_O_2_ or pretreated with KEAP1 siRNA prior to H_2_O_2_ disposition is determined by CCK8 assay (**A**) and Resazurin-conversion assay (**B**). **C** The activity of released LDH in PIG1 cells treated by H_2_O_2_ or pretreated with KEAP1 siRNA prior to H_2_O_2_ disposition, determined by LDH release assay. **D** Annexin V-FITC/PI apoptosis assay of PIG1 cells pretreated with KEAP1 siRNA, followed by treatment with H_2_O_2_. The cell viability of PIG1 cells treated by H_2_O_2_ or pretreated with PGAM5 siRNA prior to H_2_O_2_ disposition is determined by CCK8 assay (**E**) and Resazurin-conversion assay (**F**). **G** The activity of released LDH in PIG1 cells treated by H_2_O_2_ or pretreated with PGAM5 siRNA prior to H_2_O_2_ disposition, determined by LDH release assay. **H** Annexin V-FITC/PI apoptosis assay of PIG1 cells pretreated with PGAM5 siRNA, followed by treatment with H_2_O_2_. ^*^P < 0.05, ^**^P < 0.01, ^***^P < 0.001.

Additionally, the expression of KEAP1 and PGAM5 were increased in PIG1 cells after the treatment of H_2_O_2_, and employing Z-VAD and Nec-1 in the pretreatment of PIG1 cells did not alter the expression of KEAP1 and PGAM5 as indicated by Western-blot (Fig. 3C). The result was further confirmed in the lesional skin of vitiligo patients as detected by immunofluorescence assay when compared with healthy controls (Fig. 4A, B). Subsequently, the effect of the upregulation of KEAP1 and PGAM5 on melanocytes survival was assessed. We next knocked down KEAP1 and PGAM5 in PIG1 cells before the treatment with H_2_O_2_, and performed a series of the cell-survival assays. The expression of KEAP1 and PGAM5 were significantly downregulated by the corresponding siRNA (Supplementary Fig. 2A, B). The results illustrated that cellular depletion of KEAP1 significantly increased the cell viability and decreased the cytotoxicity induced by H_2_O_2_ (Fig. 5A–D). Similarly, depleting PGAM5 significantly rescued PIG1 cells from H_2_O_2_-induced death (Fig. 5E–H). Taken together, these data suggested that KEAP1 and PGAM5 play a critical role in oxidative stress-induced melanocytes death.

### Oxeiptosis is activated in melanocytes under oxidative stress

**Fig. 6:**
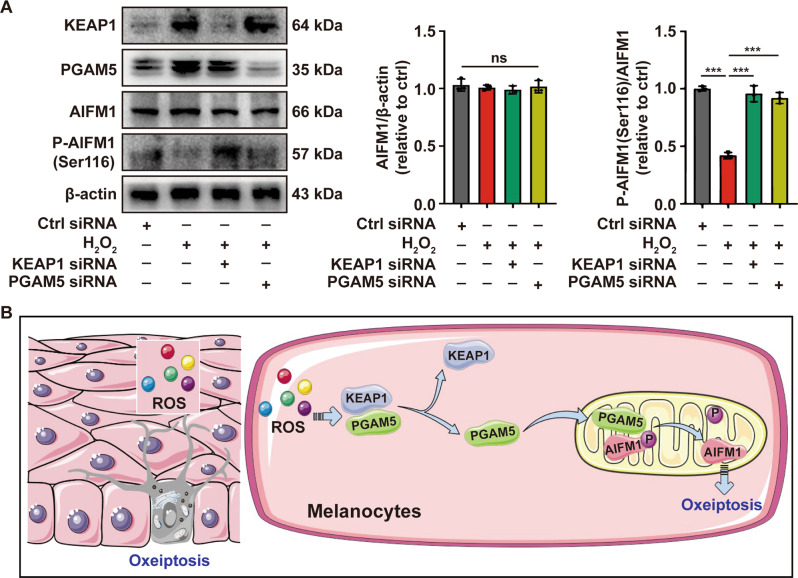
Oxeiptosis is activated by oxidative stress in melanocytes. **A** The analysis of AIFM1 phosphorylated at Ser116 in PIG1 cells pretreated with KEAP1 siRNA or PGAM5 siRNA, followed by treatment with H_2_O_2_, determined by Western-blot assay. **B** The diagram of the oxeiptosis in melanocytes under oxidative stress. ^***^P < 0.001, ns: not significant.

We next sought to identify whether the KEAP1-PGAM5-AIFM1 pathway was activated in melanocytes under oxidative stress. As shown, the depletion of KEAP1 or PGAM5 in PIG1 cells significantly reversed the dephosphorylation of AIFM1 at Ser116 induced by H_2_O_2_ (Fig. 6A). Collectively, we concluded that PGAM5 was released from KEAP1 under oxidative stress and then dephosphorylated AIFM1 at Ser116, which triggered the oxeiptosis (Fig. 6B).

## Discussion

Oxidative stress-induced melanocytes demise is central to vitiligo pathogenesis [2]. Multiple death modalities could be triggered by oxidative stress in vitiligo, including apoptosis, necroptosis, and necrosis if the redox disequilibrium is too harsh [19, 20]. In the present study, we initially found that other death modalities of H_2_O_2_-challenged melanocytes existed in addition to apoptosis and necroptosis. Further exploration indicated that AIFM1 was dephosphorylated under oxidative stress and the dephosphorylation was modulated by KEAP1 and PGAM5. Thus, melanocytes exhibited the activation of the KEAP1-PGAM5-AIFM1 signaling pathway under oxidative stress, which was a hallmark of a regulated cell death called oxeiptosis.

KEAP1 is an important sensor of oxidative stress. Physiologically, KEAP1 ubiquitinates and degrades Nrf2. The degradation of Nrf2 is impaired in oxidative stress conditions, which leads to the accumulation and nuclear translocation of Nrf2 and expression of antioxidant response element (ARE) [23]. Previous studies revealed that high-mobility group protein B1 (HMGB1) released by melanocytes under oxidative stress might perturb the Nrf2-ARE signaling pathway which further instigates apoptosis [24]. While in the present study, the function of KEAP1 was switched to disassociate PGAM5 to activate melanocytes oxeiptosis.

PGAM5 is functionally pleiotropic on account of its complex conformation. PGAM5 binds to KEAP1 by a conserved NxESGE motif formed by amino acids 77–82 [25]. Besides, PGAM5 regulates mitochondrial dynamics (e.g., mitochondrial movement, fission, and mitophagy) and programmed cell death through protein–protein interactions and its specific Ser/Thr/His protein phosphatase activity. In the face of mild mitochondrial injuries, PGAM5 activates mitochondrial biosynthesis and mitophagy as compensative reactions to restore cell viability [17]. Also, amino acids 125–156 mediate the interactions between anti-apoptotic protein Bcl-xL, which ameliorates myocardial I/R injury-induced apoptosis in cardiomyocytes [26]. When the mitochondria are severely compromised, PGAM5 might instigate excessive mitochondrial fission and abnormal mitochondrial movement, the cascade amplification of which might finally be translated into cell apoptosis and necroptosis [26, 27]. In the present study, the Ser protein phosphatase activity of PGAM5 mediated the dephosphorylation of AIFM1 in H_2_O_2_-treated melanocytes.

AIFM1 is a multifunctional cell death effector. The mitochondria-associated AIFM1 mainly consists of two pools, the larger one of which is compartmentalized in the intermembrane space (IMS) of mitochondria and attached to the inner membrane; and the smaller one (putatively 30%) loosely tethers to the cytoplasmic side of the outer mitochondrial membrane [28, 29]. In the context of apoptosis, AIFM1 is truncated to the apoptogenic form and liberated into the cytoplasm from the IMS to enter the nucleus and induce DNA degradation, though the precise mechanisms of the AIFM1 liberation remain elusive [30, 31]. In the context of parthanatos (a caspase-independent PARP-1-dependent cell death), however, the pathologically lengthened and branched PAR polymer triggers the disassociation and nuclear translocation of the outer membrane pool of AIFM1 [32], which further confers the large-scale fragmentation of DNA [33]. In the present study, we found that AIFM1 was dephosphorylated at Ser116, it remains to be delineated how the dephosphorylated AIFM1 further facilitated oxeiptosis. It was found that the dephosphorylated AIFM1was sustained in the mitochondria and relocated to circular structures within mitochondria [14]. The compartmentalization of AIFM1 in mitochondria was previously described only in *Drosophila melanogaster* due to the deficiency of the canonical proline/glutamic acid/serine/threonine-rich (PEST) sequence motif, which mediates the truncation and liberation of AIFM1 to the cytoplasm, but the PEST motifs keep integrity in mammals [34–36].

The immune consequence of melanocytes oxeiptosis is well worth pursuing. Immune consequences of melanocytes death like inflammation and auto-antigens exposure are always discussed in vitiligo pathogenesis. Ozone-exposed PGAM5^−/−^ mice exhibited enhanced expression levels of cytokines and chemokines like interleukin-6 (IL-6) and CXCL, chemokine (C-X-C motif) ligand 1 (CXCL1), which indicated that PGAM5 might suppress the inflammation instigated by ozone or virus exposure and that oxeiptosis might not be a pro-inflammatory cell death modality [13]. Although melanocytes oxeiptosis might be anti-inflammatory, the melanocytic auto-antigens exposure triggered by oxeiptosis is not ruled out. Cell modalities might be categorized as pro- or anti-inflammatory, while the immunogenicity of the dead cell is independent of cell modalities [37]. Therefore, melanocytes undergoing oxeiptosis in oxidative stress is probably immunogenic, which is yet to be fully elucidated.

At least three death modalities, including apoptosis, necroptosis, and oxeiptosis, might exist in melanocytes undergoing oxidative stress as of yet, but their respective proportions and contributions in vitiligo pathogenesis are still incompletely understood. Besides, further in vivo studies should be performed to delineate the immune consequences of melanocytes oxeiptosis in vitiligo.

The present study illustrated that melanocytes might undergo oxeiptosis that was mediated by the KEAP1-PGAM5-AIFM1 signaling pathway under oxidative stress. The oxeiptosis of melanocytes might also be a therapeutically exploitable cellular event in vitiligo treatment.

## Materials and methods

### Cell culture and treatment

The immortalized normal human epidermal melanocyte cell line PIG1 (a gift from Dr. Caroline Le Poole, Loyola University Chicago, Maywood, IL, USA) was cultured in Medium 254 (Invitrogen, Portland, USA) supplemented with human melanocyte growth supplements (#1828072, Gibco, USA), 5% fetal bovine serum (#04-001-1ACS, Biological Industries, Israel), and penicillin–streptomycin antibiotic mix at 37 °C in the presence of 5% CO_2_. Caspase inhibitor Z-VAD (#S7023, Selleckchem, USA) and necroptosis inhibitor Nec-1 (#S8037, Selleckchem, USA) were used to pretreat cells for 1 h. Oxidative stress was induced by the treatment with hydrogen peroxide (H_2_O_2_) (#316989, Sigma-Aldrich, USA) for 24 h at different concentrations. Small interfering RNA targeting KEAP1 (KEAP1 siRNA) (#5289, Cell Signaling Technology, USA) or PGAM5 (PGAM5 siRNA) (5′-CGGCCGUGGCGGUAGGGAATT-3′, GenePharma, China) were transfected into cells according to the manufacturer’s recommendations.

### Patients and samples

The skin samples of vitiligo were obtained from patients given a diagnosis of vitiligo in the Department of Dermatology of Xijing Hospital. The skin samples of age- and sex-matched healthy control were collected from individuals who underwent double eyelid surgery in the department of plastic and cosmetic surgery of Xijing Hospital. The samples were embedded in paraffin at 4 °C. All participants were informed, consent, and signed a written consent form. Protocols were designed and performed according to the principles of the Declaration of Helsinki and were approved by the ethics review board of Fourth Military Medical University.

### CCK8 assay

CCK8 assay was used to detect cell viability. PIG1 cells were seeded into 96-well plates at a density of 8000 per well. After corresponding treatments, cells were incubated with 100 μl fresh medium with 10 μl CCK8 solution (#C008, 7Sea biotech, China) for 2 h at 37 °C, and then the optical density (OD) was measured at 450 nm by Model 680 Microplate Reader (Bio-Rad, USA).

### Resazurin-conversion assay

Resazurin-based cell viability assay was used to measure general enzyme reduction activity. PIG1 cells were seeded into 96-well plates at the density of 8000 per well and cultured under the corresponding treatments. Twenty microlitre resazurin dye reagent (#QDY-002-C, RHINO BIO, China) was added to per well and incubated for 8 h at 37 °C, followed by measurement of OD at 570 nm using Model 680 Microplate Reader (Bio-Rad, USA).

### Lactate dehydrogenase (LDH) release assay

LDH release assay was used to detect the activity of LDH released during cell injury. PIG1 cells were plated into 96-well plates at the density of 8000 cells per well and treated with corresponding treatments. The LDH levels in the supernatant were detected using the LDH Cytotoxicity Assay kit ((#C0016, Beyotime Biotechnology, China) according to the manufacturer’s recommendations. The OD was measured at 490 nm by Model 680 Microplate Reader (Bio-Rad, USA).

### Annexin V-FITC/propidium iodide (PI) apoptosis assay

PIG1 cells were plated into 6-well plates at the density of 3 × 10^5^ cells per well and were treated with corresponding treatments. Cell apoptosis was detected by the Annexin V-FITC/PI cell apoptosis kit (#A005, 7Sea biotech, China). Simply, the PIG1 cells were collected and resuspended in a 400 μl binding buffer, followed by the addition of 5 μl of Annexin V-FITC and 10 μl PI. The mixture was incubated for 15 min at room temperature in the dark. The apoptosis rates were measured by flow cytometry (FC500, Beckman Coulter, Miami, Fl, USA) and analyzed with Expo32 software (Beckman Coulter, USA).

### Western-blot assay

After corresponding treatments, the cells were washed three times with ice-cold PBS and harvested, and then lysed with RIPA lysis buffer (#P0013C, Beyotime Biotechnology, China), which contains cocktails of protease inhibitor and phosphatase inhibitor, for 15 min on ice followed by centrifugation at 12,000 rpm for 10 min. Each protein sample was quantified using the BCA Protein Assay kit (#PA115, TianGen, China). Equal amounts of protein samples were separated by 10% sodium dodecyl sulfate-polyacrylamide gel electrophoresis (Bio-Rad, USA) and transferred to Polyvinylidene difluoride membranes (Millipore, Billerica, USA). Page Ruler Plus Prestained Protein Ladder (Fermentas, Hanover, USA) was used to confirm protein electrophoresis and transfer. Then the phosphorylation form of the molecule was blocked with 5% bovine serum albumin, while the total form of the molecule was blocked with 5% non-fat dry milk for 1 h at room temperature. After washed with TBST (TBS + 0.1% [v/v] Tween-20) transitorily, the membranes were incubated with primary antibodies against β-actin (8H10D10) (1:5000, #3700, Cell Signaling Technology, USA), MLKL (D2I6N) (1:1000, #14993, Cell Signaling Technology, USA), phospho-MLKL (Ser-358) (D6H3V) (1:1000, #91689, Cell Signaling Technology, USA), Caspase3 (1:1000, #9662, Cell Signaling Technology, USA), Cleaved-caspase3 (Asp175) (1:1000, #9661, Cell Signaling Technology, USA), AIFM1 (1:1000, #5318, Cell Signaling Technology, USA), phospho-AIFM1 (Ser-116) (1:1000, #AP5501, ECM Biosciences, USA), KEAP1 (D6B12) (1:1000, #8047, Cell Signaling Technology, USA) and PGAM5 (1:1000, #ab126534, Abcam, UK) at 4 °C overnight. After extensive rinsing, the membranes were incubated with corresponding secondary antibodies (Goat Anti-Rabbit IgG Antibody, Peroxidase Conjugated, 1:5000, #AP132P, Sigma-Aldrich, USA; Goat Anti-Mouse IgG Antibody, Peroxidase Conjugated, 1:5000, #AP124P, Sigma-Aldrich, USA) for 1 h at room temperature. The bands were detected with an enhanced chemiluminescence reagent western blotting detection system and quantified using Image J software. (#871BRO7308, Bio-Rad, USA).

### Immunofluorescence assay

For cell immunofluorescence assay, the PIG1 cells were cultured on laser confocal glass dishes (#801002, NEST Biotechnology, China) at 1.0 × 10^5^ cells per dish. After corresponding treatments, the cells were washed three times with phosphate buffer solution (PBS) and fixed with 4% paraformaldehyde for 10 min, followed by incubation with 0.1% Triton X-100 for 10 min and blocked with normal goat serum for 30 min. Then, cells were incubated with primary antibody (KEAP1 (D6B12) Rabbit mAb, 1:200, #8047, Cell Signaling Technology, USA; PGAM5 Rabbit mAb, 1:200, #ab126534, Abcam, UK; COX-IV Mouse mAb, 1:200, #ab33985, Abcam, UK) at 4 °C overnight. The corresponding secondary antibody (Goat anti-Rabbit IgG Green 488, 1:200, #ab150077, Abcam, UK; Goat anti-Mouse IgG Red Cy3, 1:200, #ab97035, Abcam, UK) was incubated for 1 h at room temperature in the dark. Then the cell nucleus was marked with the nuclear dye 4′,6-diamidino-2-phenylindole (DAPI) (1:1000, #ab104139, Abcam, UK) for 10 min at room temperature in the dark. Cells were washed three times with PBS after each step. The fluorescence was detected by laser confocal microscopy (#LSM510, Carl Zeiss AB, Germany). The quantitative analysis of co-localization between KEAP1 or PGAM5 and mitochondria was evaluated by calculating the ratio of KEAP1^+^COX IV^+^ dots or PGAM5^+^COX IV^+^ dots to COXIV^+^ dots. Three cells were randomly selected from each group and the experiment was duplicated three times for statistical analysis.

For skin specimens, paraffin-embedded 5-μm tissue sections were deparaffinized and heat-mediated antigen retrieval with Tris-EDTA buffer (PH = 9.0). Next, the skin sections were incubated with 5% goat serum (#AR0009, BosterBio, USA) for 30 min at room temperature. The remaining procedures were similar to the cell immunofluorescence assay with the use of an additional primary anti-Melan A (1:200, #ab187369, Abcam, UK) and the corresponding secondary Goat anti-Rabbit IgG Red Cy3 (1:200, #ab6939, Abcam, UK).

### Statistical analysis

Each experiment was performed at least three times. GraphPad Prism 7.0 software for Windows (USA) was used to perform all the statistical analyses. All data were shown as mean ± SD. One-way analysis of variance was used to analyze the experiments with multiple groups, and Tukey’s multiple comparisons test was used to compare the mean of each group with the mean of every other group. ^*^P < 0.05, ^**^P < 0.01, ^***^P < 0.001, ns: not significant.

## Supplementary information

Supplementary figures

Supplementary original data for western-blot assay

Original data set for apoptosis assay

Original data set for immunofluorescence assay

## Data Availability

The full and uncropped Western-blot figures are available from Supplementary material. The raw data and detailed materials and methods are available from the corresponding author on reasonable request.
