# Supplementary material for: Oxeiptosis: a novel pathway of melanocytes death in response to oxidative stress in vitiligo
Source: Cell Death Discov. 2022 Feb 17;8:70. doi: 10.1038/s41420-022-00863-3 (PMC8854565; doi:10.1038/s41420-022-00863-3)

**Supplementary figure 1. Z-VAD and Nec-1 effectively inhibited oxidative stress-induced apoptosis and necroptosis of melanocyte.** The expression of P-MLKL and Cleaved-caspase3 in PIG1 cells treatment with Z-VAD and Nec-1 prior to H_2_O_2_ disposition were detected by Western-blot assay. ^**^*P*＜0.01, ^***^*P*＜0.001, ns: not significant.


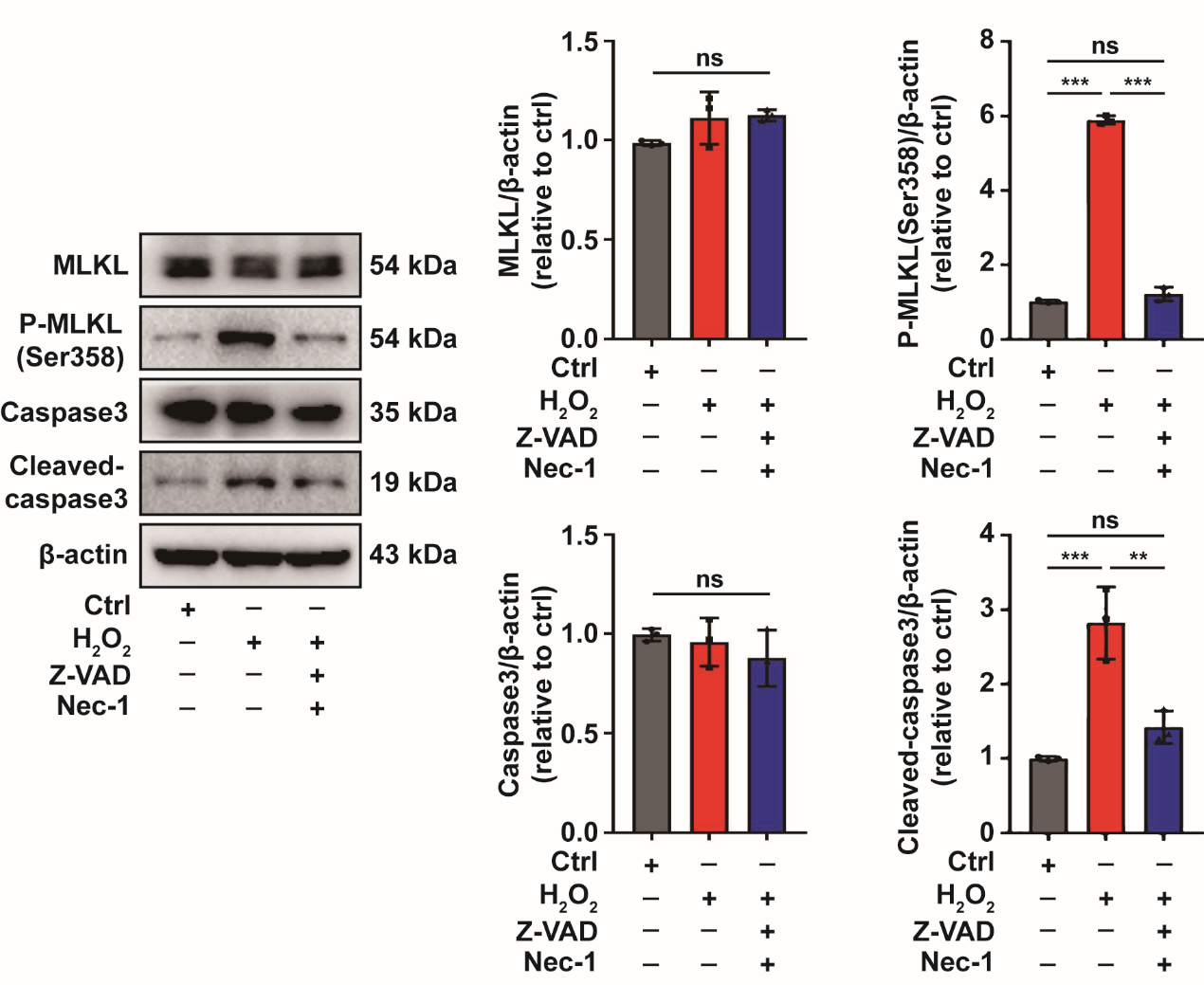


**Supplementary figure 2. The expression of KEAP1 and PGAM5 were decreased by corresponding siRNA.** The expression of KEAP1 **(A)** and PGAM5 **(B)** in PIG1 cells treatment with KEAP1 siRNA or PGAM5 siRNA were detected by Western-blot assay. ^***^*P*＜0.001.


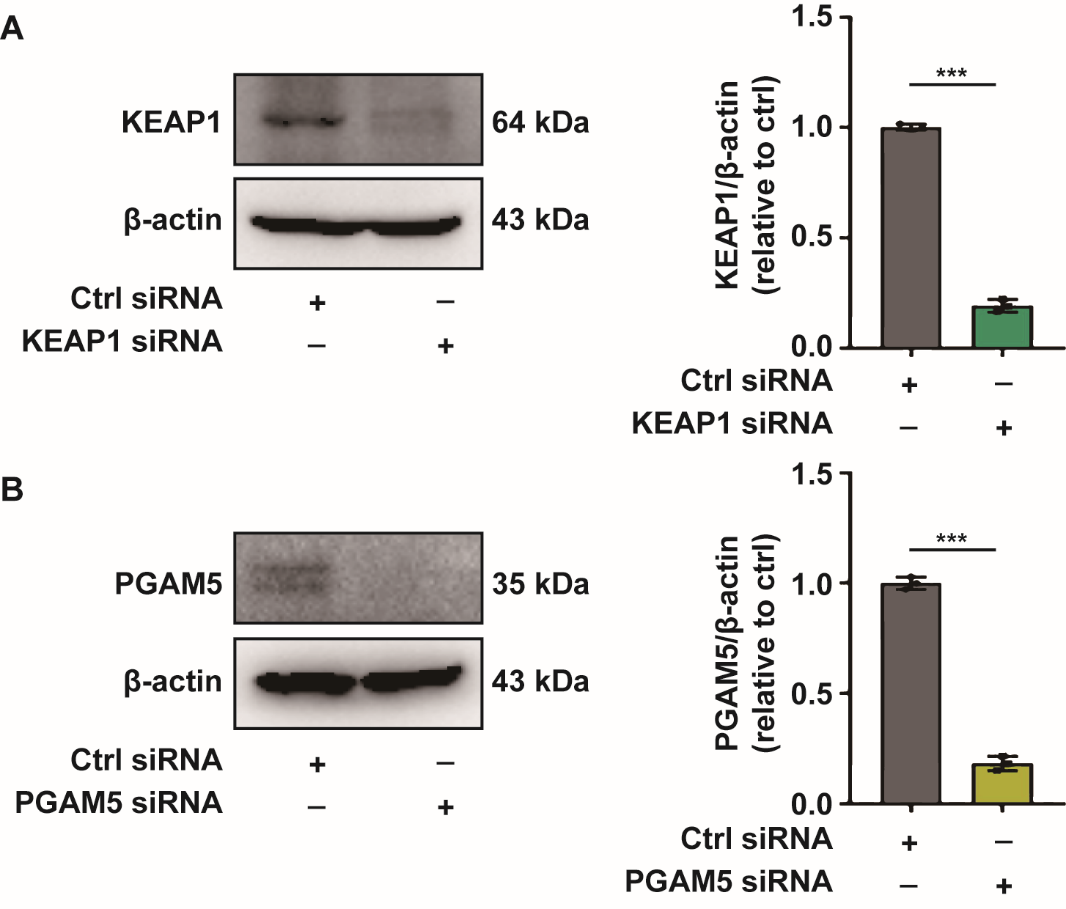

Supplement: Supplementary file 1 — Supplementary figures [file 41420_2022_863_MOESM1_ESM.docx]
