# Supplementary material for: Oxeiptosis: a novel pathway of melanocytes death in response to oxidative stress in vitiligo
Source: Cell Death Discov. 2022 Feb 17;8:70. doi: 10.1038/s41420-022-00863-3 (PMC8854565; doi:10.1038/s41420-022-00863-3)

**Figure 2A**

AIFM1


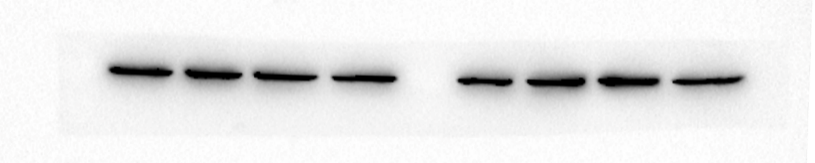


P-AIFM1(Ser116)


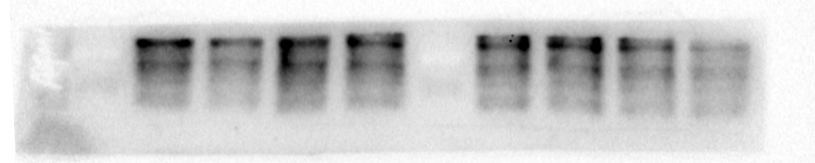


β-actin


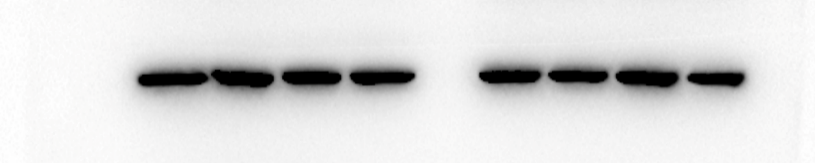


**Figure 2B**

AIFM1


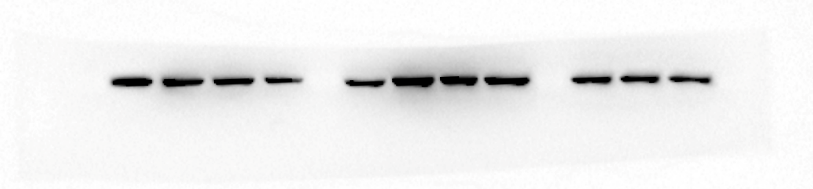


P-AIFM1(Ser116)


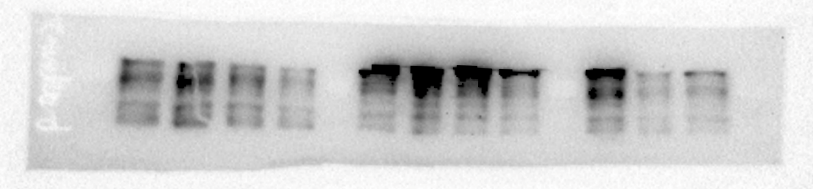


β-actin


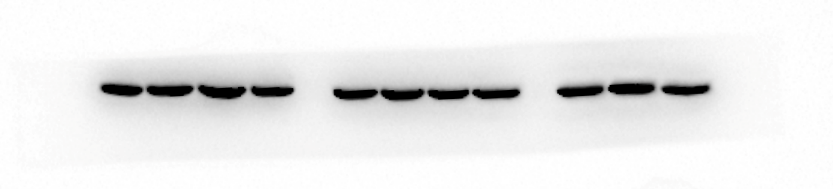


**Figure 3C**

KEAP1


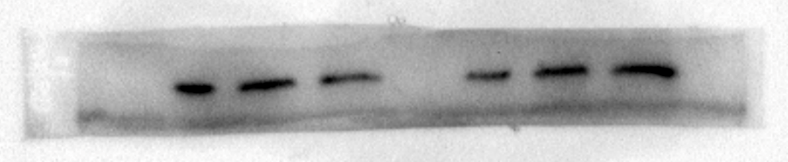


PGAM5


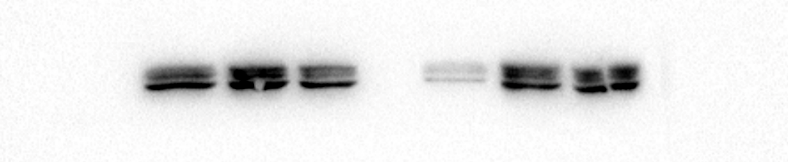


β-actin


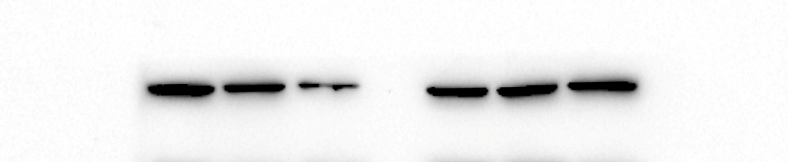


**Figure 6A**

KEAP1


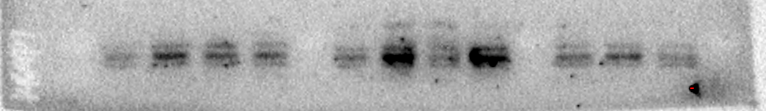


PGAM5


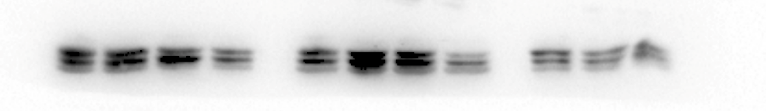


AIFM1


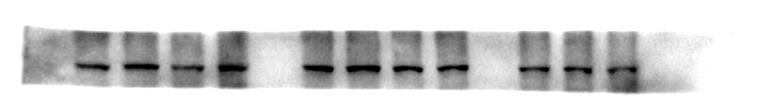


P-AIFM1(Ser116)


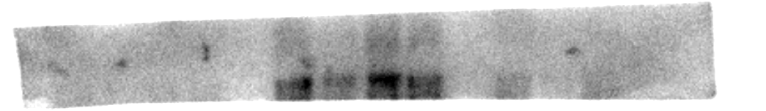


β-actin


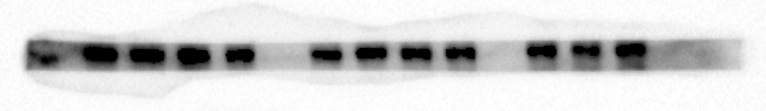


**Supplementary figure 1**

MLKL


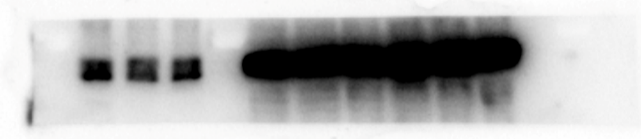


P-MLKL(Ser358)


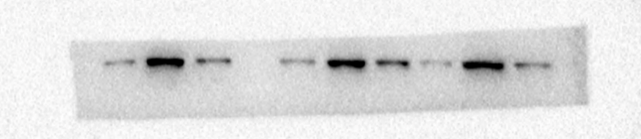


Caspase3


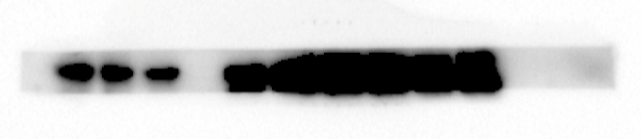


Cleaved-caspase3


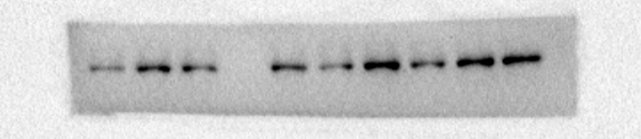


β-actin


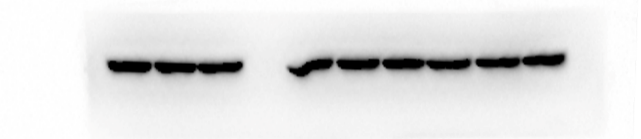


**Supplementary figure 2A**

KEAP1


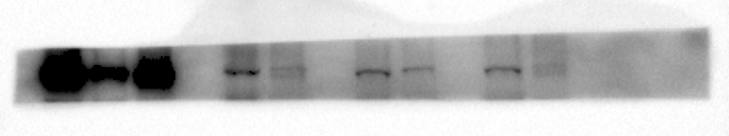


β-actin


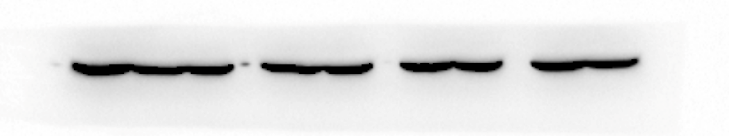


**Supplementary figure 2B**

PGAM5


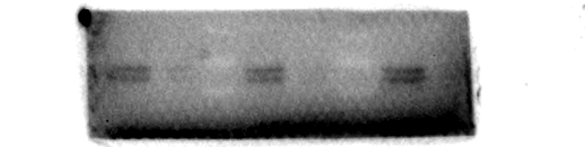


β-actin


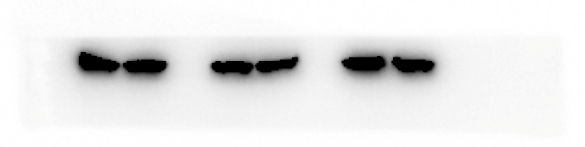

Supplement: Supplementary file 2 — Supplementary original data for western-blot assay [file 41420_2022_863_MOESM2_ESM.docx]
